# Embryonic expression of shuttle craft, a Drosophila gene involved in neuron development, is associated with adult lifespan

**DOI:** 10.18632/aging.100712

**Published:** 2014-12-28

**Authors:** Natalia V. Roshina, Alexander V. Symonenko, Anna V. Krementsova, Mikhail V. Trostnikov, Elena G. Pasyukova

**Affiliations:** ^1^ Institute of Molecular Genetics of Russian Academy of Sciences, Moscow, 123182, Russia; ^2^ Emmanuel Institute of Biochemical Physics of Russian Academy of Sciences, Moscow, 119334, Russia

**Keywords:** lifespan, aging, embryonic gene expression, locomotion, reproduction, synaptic function, Drosophila melanogaster

## Abstract

Despite the progress in aging research that highlights the role of the nervous system in longevity, whether genes that control development and consequently structure of the nervous system affect lifespan is unclear. We demonstrated that a mutation in *shuttle craft*, a gene involved in the nervous system development, increased the lifespan of unmated females and decreased the lifespan of mated females, without affecting males. Precise reversions of the mutation lead to the restoration of the lifespan specific to control females. In mutant unmated females, increased lifespan was associated with elevated locomotion at older ages, indicating slowed aging. In mutant mated females, reproduction was decreased compared to controls, indicating a lack of tradeoff between this trait and lifespan. No differences in *shuttle craft* transcription were observed between whole bodies, ovaries, and brains of mutant and control females of different ages, either unmated or mated. The amount of *shuttle craft* transcript appeared to be substantially decreased in mutant embryos. Our results demonstrated that a gene that regulates development of the nervous system might also influence longevity, and thus expanded the spectrum of genes involved in lifespan control. We hypothesize that this “carry-over” effect might be the result of transcription regulation in embryos.

## INTRODUCTION

Aging and longevity are intimately associated with functional activity and overall nervous system status. The nervous system has long been suggested as a key tissue that defines lifespan. The influence of the nervous system on lifespan was initially indicated by an observation that increased expression of some genes exclusively in the nervous tissue of transgenic animals resulted in increased lifespan [1, 2]. Later, this finding was supported by the discovery of multiple molecular mechanisms for the impact of the nervous system on lifespan [for review, see 3, 4]. Despite this progress, little is known about how genes that control development and consequently structural properties of the nervous system affect normal lifespan.

The nervous system is important for processing complex information from internal and external sources, which strongly affects aging and longevity of animals. Accordingly, the functionality of the nervous system is crucial for survival. Structural and functional fitness of the nervous system is largely determined by the allelic composition of genes. Earlier, we demonstrated that several genes that encode RNA polymerase II transcription factors and are involved in development of the nervous system affect lifespan variation in Drosophila melanogaster [5-7]. This article presents the results of further study of one of these genes, shuttle craft (stc).

*stc* encodes an RNA polymerase II transcription factor homologous to human transcription factor NF-X1 [8]. STC protein contains seven copies of a cysteine-rich motif that determines binding specificity to conserved X-box sequence that is present in a variety of eukaryotic genes. In *Drosophila*, *stc* is expressed throughout all developmental stages and in adults. In embryos, *stc* is expressed in the central nervous system, where it is required to maintain the proper morphology of motoneuronal axon nerve routes [8]. *stc* mutations are lethal at the end of embryogenesis because of an inability of mutants to coordinate the peristaltic muscular contractions required for hatching. In adults, *stc* expression is highest in ovaries and provides essential maternal contributions to early development: embryos deprived of a maternal source of STC show abnormal development of the ventral nerve cord and misguided migration of motoneuronal axons [9].

In this paper, we demonstrate that a viable *stc* mutation caused by inserting of a vector construct into the untranslated region of the gene affected lifespan of flies in a sex-specific manner. Four independent reversions of this mutation were accompanied by reversions in lifespan phenotype. In mutant virgin females, both survival curves and age-dependent changes in locomotion indicated that mutation increased lifespan and slowed aging. In mutant mated females, lifespan and reproduction were decreased compared to controls, indicating a lack of tradeoff between these traits. The amount of *stc* transcript appeared to be substantially increased in mutant embryos but not in larvae and adult females of any age, whether virgin or mated. This result led us to hypothesize that lifespan might depend on gene function during early development.

## RESULTS

### Reversions

**Figure 1:**
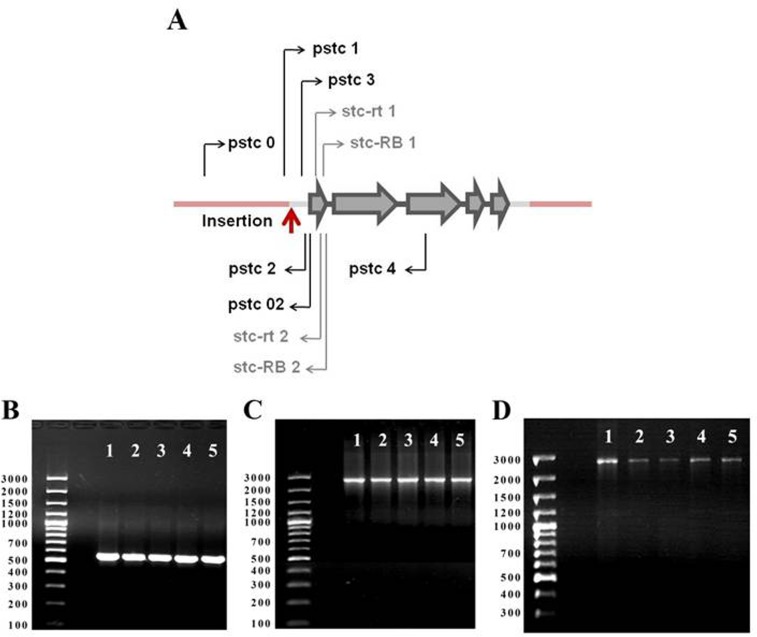
*stc* gene map and PCR analysis of *stc* structure in revertant lines. (**A**) *stc* gene map. Gray arrows: exons; black sections:introns; gray sections: untranslataed regions; pink sections: background sequences; red arrow: *P{SUPor-P}stc^KG01230^* insertion site; black arrows: primers used for PCR; gray arrows: primers used for Real Time RT-qPCR. (**B**) PCR with primers pstc1 and pstc2 (expected product size 555 bp); (**C**) PCR with primers pstc0 and pstc02 (expected product size 2508 bp); (**D**) PCR with primers pstc3 and pstc4 (expected product size 2562 bp). 1: control line; 2: rev1, 3: rev3, 4: rev4; 5: rev5.

A control line (control) with the genotype w1118 and a line with the stc mutation, w1118; P{SUPor-P}stcKG01230 (stcP, Figure 1A) were used in this study. Four lines with reversions of the w+ marker phenotype (rev1, rev3, rev4, rev5) were obtained from stcP using standard substitution crosses with balancer chromosomes and the delta 2-3 source of P element transposase [10]. For each line, PCR with primers surrounding the site of the initial P{SUPor-P} insertion were used to assess the nature of reversions. PCR fragment sizes were identical in the control line and in all lines with reversions (Figure 1B), indicating precise excisions of the P{SUPor-P} construct. This was further confirmed by sequencing PCR fragments: all sequences were identical to the standard gene sequence (http://flybase.org). To confirm that excisions did not cause regional aberrations, two additional PCR reactions with primers that amplified approximately 2.5 kb on both sides of the insertion site were made for each line; no deviations from the control line were observed in any line (Figures 1C, D). Thus, in all four lines, the vector construct was precisely excised from the insertion site and complete restoration of the original gene structure was achieved. Negative results were obtained for all six lines (control, stcP, rev1, rev3, rev4, rev5) in a test for the presence of Wolbachia, a Drosophila symbiont known to affect life history traits [11].

### Lifespan

**Figure 2:**
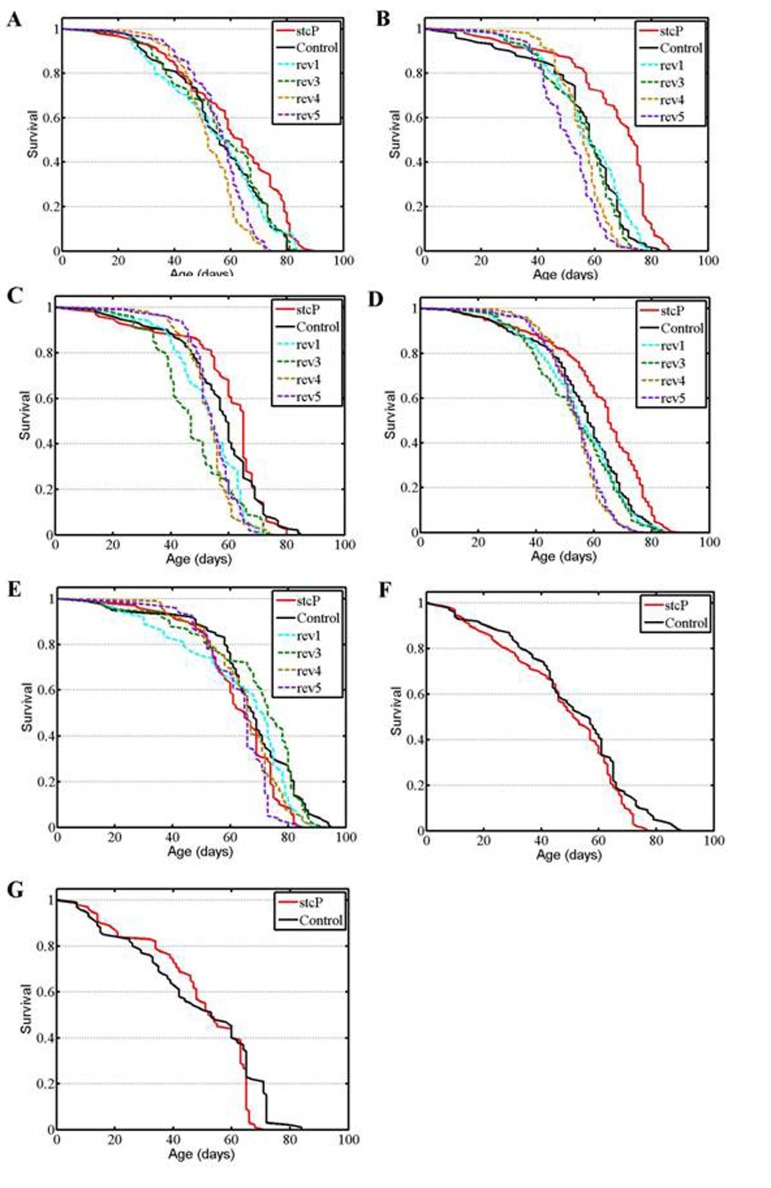
Survival of unmated females and males. (**A, B, C**) experiments #1, #2, #3 with females. (**D**) combined data for the three experiments. (**E, F, G**) experiments #1, #2, #3 with males.

**Figure 3:**
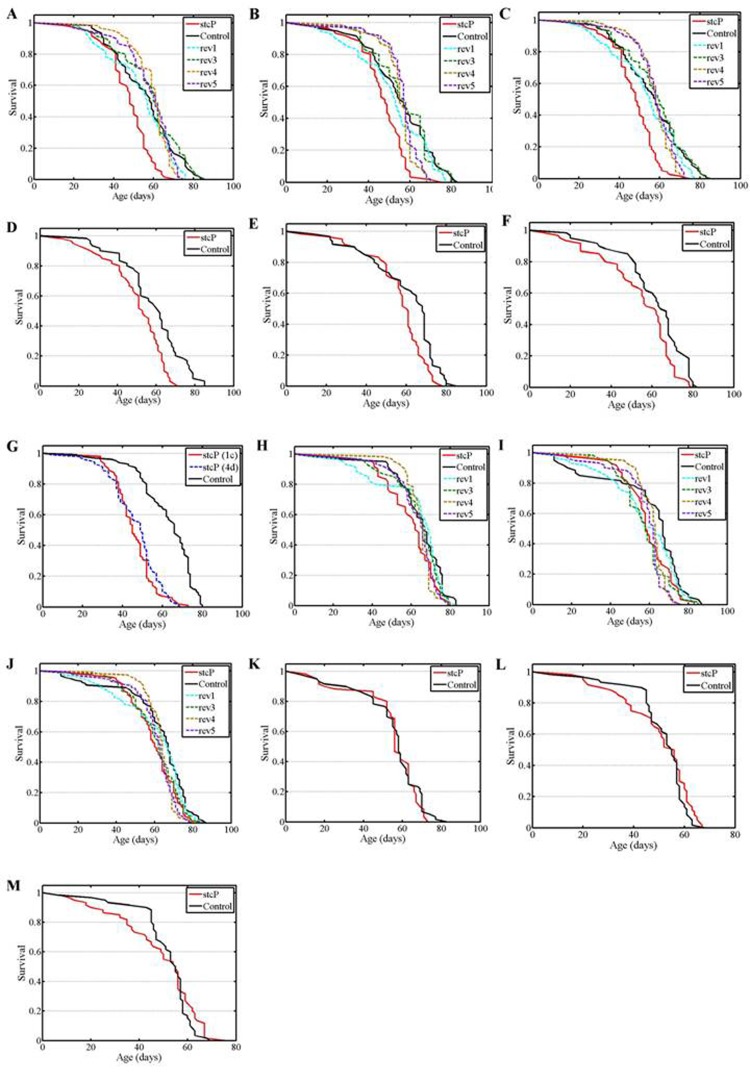
Survival of mated females and males. (**A, B**) experiments #1, #2 with females. (**C**) combined data for experiments #1, #2 with females. (**D, E, F, G**) experiments #3, #4, #5, #6 with females. H, I: experiments #1, #2 with males. J: combined data for experiments #1, #2 with males. (**K, L, M**) experiments #3, #4, #5 with males.

We assessed the effect of the *stc^KG01230^* mutation on lifespan relative to control and reversion lines in unmated and mated females and males. For each experiment, we calculated the following parameters: mean lifespan, median lifespan, minimum and maximum lifespan, lifespan lower and upper quartiles, lifespan of the 10th and 90th percentiles; variance, standard deviation, and standard error for the mean lifespan (Table 1). Figures 2, 3 show survival curves.

**Table 1:** Distributive statistics of the lifespan

Exp. No	Line	N	Mean	Median	Minimum	Maximum	Lower Quartile	Upper Quartile	Percentile 10	Percentile 90	Variance	Standard Deviation	Standard Error	P values for comparisons with stcP line		
														t-test	Kruskal-Wallis test	Kolmogor ov-Smimov test
**Unmated females**																
1	Control	100	56	56	18	82	46	70	30	77	276.48	16.63	1.66	***0.0488***	***0.0224***	***0.0366***
	stcP	100	61	64	11	90	46	78	35	81	346.83	18.62	1.86			
	rev1	100	54	58	19	87	39	68	28	75	320.1	17.89	1.79	***0.018***	***0.0143***	***0.0158***
	rev3	100	57	59	10	85	41	70	32	77	298.8	17.29	1.73	*0.1126*	*0.0533*	***0.0243***
	rev4	100	52	52	24	72	46	60	38	66	105.89	10.29	1.03	***0.0001***	***0.0001***	***0.0001***
	rev5	100	55	58	18	74	50	63	40	68	124.33	11.15	1.12	***0.0191***	***0.0042***	***0.0001***
2	Control	100	56	58	4	83	53	68	31	71	265.65	16.3	1.63	***0.0001***	***0.0001***	***0.0001***
	stcP	100	66	74	14	87	58	77	44	81	277.02	16.64	1.66			
	rev1	100	57	58	10	79	48	68	40	75	202.44	14.23	1.42	***0.0001***	***0.0001***	***0.0001***
	rev3	100	55	59	17	75	46	64	38	70	161.35	12.7	1.27	***0.0001***	***0.0001***	***0.0001***
	rev4	100	55	56	27	76	50	61	46	66	73.04	8.55	0.85	***0.0001***	***0.0001***	***0.0001***
	rev5	100	51	51	20	78	42	58	38	64	113.75	10.67	1.07	***0.0001***	***0.0001***	***0.0001***
3	Control	100	56	60	14	85	50	66	39	72	220.4	14.85	1.48	*0.2564*	***0.0324***	***0.0063***
	stcP	100	59	65	7	80	55	68	34	72	239.16	15.46	1.55			
	rev1	100	52	54	13	71	45	63	38	65	146.23	12.09	1.21	***0.0006***	***0.0001***	***0.0001***
	rev3	100	48	47	15	76	39	59	34	66	179.72	13.41	1.34	***0.0001***	***0.0001***	***0.0001***
	rev4	100	53	54	27	74	49	57	42	61	67.15	8.19	0.82	***0.0005***	***0.0001***	***0.0001***
	rev5	100	54	54	26	70	50	59	46	64	63.71	7.98	0.8	***0.0045***	***0.0001***	***0.0001***
1+2+3	Control	300	56	58	4	85	49	68	31	73	252.76	15.9	0.92	***0.0001***	***0.0001***	***0.0001***
	stcP	300	62	65	7	90	55	75	37	80	295.6	17.19	0.99			
	rev1	300	55	55	10	87	45	66	33	72	225.26	15.01	0.87	***0.0001***	***0.0001***	***0.0001***
	rev3	300	53	54	10	85	41	66	34	72	226.96	15.07	0.87	***0.0001***	***0.0001***	***0.0001***
	rev4	300	53	54	24	76	47	59	41	64	83.17	9.12	0.53	***0.0001***	***0.0001***	***0.0001***
	rev5	300	53	55	18	78	47	60	40	65	104.27	10.21	0.59	***0.0001***	***0.0001***	***0.0001***
Unmated males																
1	Control	100	67	68	10	95	60	81	48	86	296.4	17.2	1.7	*0.0565*	*0.0507*	*0.0541*
	stcP	100	62	65	10	84	54	74	46	78	206.2	14.4	1.4			
	rev1	100	66	72	15	89	52	78	30	81	373.1	19.3	1.9	*0.1369*	***0.0018***	***0.0001***
	rev3	100	68	73	11	93	58	81	39	85	340.6	18.5	1.8	***0.0197***	***0.0004***	***0.0001***
	rev4	100	65	66	34	88	58	72	46	79	164.2	12.8	1.3	*0.2479*	*0.3956*	*0.281*
	rev5	100	62	66	15	85	55	72	47	73	135.9	11.7	1.2	*0.9097*	*0.4274*	***0.0063***
2	Control	100	51	57	4	89	40	65	22	76	402.5	20.1	2	0.2194	0.3035	0.5806
	stcP	100	48	52	5	77	33	64	16	70	377.2	19.4	1.9			
3	Control	100	48	53	7	84	33	65	14	72	471.9	21.7	2.2	0.854	0.741	0.2243
	stcP	100	49	53	6	71	40	65	17	65	324.3	18	1.8			
Mated females																
1	Control	60	56	59	29	84	44	67	35	76	221.3	14.9	1.9	***0.0004***	***0.0009***	***0.0003***
	stcP	60	48	48	15	71	41	55	32	61	121.7	11	1.4			
	rev1	60	54	57	19	76	43	67	29	71	241.1	15.5	2	***0.0134***	***0.0037***	***0.0013***
	rev3	60	58	61	23	86	49	71	37	78	241.6	15.5	2	***0.0001***	***0.0001***	***0.0001***
	rev4	60	59	61	32	73	54	66	47	68	82	9.1	1.2	***0.0001***	***0.0001***	***0.0001***
	rev5	60	58	62	18	75	51	68	42	72	162.7	12.8	1.6	***0.0001***	***0.0001***	***0.0001***
2	Control	60	55	57	10	83	45	67	36	74	257.4	16	2.1	***0.0017***	***0.0011***	***0.0013***
	stcP	60	47	49	9	75	41	55	34	58	132.8	11.5	1.5			
	rev1	60	52	53	11	77	42	68	25	71	298.6	17.3	2.2	*0.0848*	***0.0435***	***0.009***
	rev3	60	57	57	28	82	45	67	36	74	194.2	13.9	1.8	***0.0001***	***0.0002***	***0.0001***
	rev4	60	55	56	28	68	52	59	45	65	68.1	8.3	1.1	***0.0001***	***0.0001***	***0.0001***
	rev5	60	56	58	10	70	54	60	47	66	86.2	9.3	1.2	***0.0001***	***0.0001***	***0.0001***
1+2	Control	120	56	58	10	84	44	67	35	76	237.6	15.4	1.4	***0.0001***	***0.0001***	***0.0001***
	stcP	120	47	48	9	75	41	55	33	60	126.3	11.2	1			
	rev1	120	52	55	11	77	42	67	28	71	268.6	16.4	1.5	***0.0031***	***0.0004***	***0.0001***
	rev3	120	58	60	23	86	47	67	36	77	216.6	14.7	1.3	***0.0001***	***0.0001***	***0.0001***
	rev4	120	57	58	28	73	54	63	46	68	79.1	8.9	0.8	***0.0001***	***0.0001***	***0.0001***
	rev5	120	57	58	10	75	53	65	44	69	124	11.1	1	***0.0001***	***0.0001***	***0.0001***
3	Control	60	59	62	24	85	51	69	36	79	245.6	15.7	2	**0.0029**	**0.0041**	**0.0162**
	stcP	60	50	53	10	71	42	61	27	65	211.6	14.5	1.9			
4	Control	60	60	68	13	85	50	72	35	77	295.1	17.2	2.2	0.2006	**0.0275**	**0.0025**
	stcP	60	56	61	7	78	50	66	35	72	205.1	14.3	1.8			
5	Control	60	61	66	18	82	52	72	36	78	256.8	16	2.1	**0.0295**	**0.0226**	**0.047**
	stcP	60	54	62	7	79	44	67	25	71	314.4	17.7	2.3			
6	Control	100	63	66	17	80	52	73	47	78	13.5	21.4	1.3	**0.0001; 0.0001**	**0.0001; 0.0001**	**0.0001; 0.0001**
	stcP	100	46	45	11	73	39	52	33	57	10.3	22.4	1			
			47	49	5	69	38	54	30	61	12.2	26.2	1.2			
Mated males																
1	Control	60	65	67	10	83	59	75	50	76	171.9	13.1	1.7	***0.0183***	***0.0406***	*0.0763*
	stcP	60	60	63	18	79	51	70	43	75	162.1	12.7	1.6			
	rev1	60	62	69	11	84	61	73	32	76	288	17	2.2	*0.3861*	***0.0375***	***0.0162***
	rev3	60	63	67	17	79	60	72	42	75	164.7	12.8	1.7	*0.1327*	*0.0644*	*0.0763*
	rev4	57	65	65	45	80	61	69	57	72	44.4	6.7	0.9	***0.0121***	*0.1018*	***0.02***
	rev5	60	63	66	16	80	58	70	49	73	129	11.4	1.5	*0.1957*	*0.1855*	*0.1813*
2	Control	60	60	67	11	87	58	73	20	77	429.3	20.7	2.7	*0.734*	*0.0563*	***0.0225***
	stcP	60	59	59	13	83	53	69	44	75	164.9	12.8	1.7			
	rev1	60	58	62	11	84	48	71	31	77	326.7	18.1	2.3	*0.6382*	*0.9497*	*0.3752*
	rev3	60	58	58	31	85	50	64	42	74	141.2	11.9	1.5	*0.5317*	*0.3689*	*0.6604*
	rev4	60	61	63	28	77	57	65	53	69	73.2	8.6	1.1	*0.3098*	*0.2619*	***0.047***
	rev5	60	58	62	16	76	56	63	43	69	144.9	12	1.6	*0.6501*	*0.9225*	*0.2656*
3	Control	60	55	58	6	83	52	66	33	70	263.9	16.2	2.1	0.8682	0.885	0.5095
	stcP	60	55	56	10	73	53	65	23	70	242.8	15.6	2			
4	Control	60	52	55	7	68	47	58	44	61	115.9	10.8	1.4	0.5321	0.8561	0.3752
	stcP	60	50	56	15	67	41	61	29	64	183.5	13.5	1.7			
5	Control	60	48	51	10	69	43	57	28	62	181.7	13.5	1.7	0.2596	0.6532	0.1196
	stcP	60	49	55	7	76	38	61	23	67	263.5	16.2	2.1			

Standard errors were calculated from the variance among vials.

Significant P-values are in bold case. When multiple lines were compared (P values in italics), two underscored lines indicate P values that survived False Discovery Rate correction and a single bold underscore indicates P values that survived Bonferroni correction.

A significantly increased lifespan was detected in unmated mutant females compared to control females and to females of reversion lines rev1, rev4 and rev5 (Table 1, Figure 2A). To verify these results, the experiment was repeated twice over two years. In the second and third experiments, the lifespan of mutant females was significantly higher than the lifespan of control females and females of all four reversion lines (Table 1, Figures 2B, 2C). The combined results of the three experiments (Table 1, Figure 2D) clearly demonstrated a difference in lifespan between control and mutant unmated females and between mutant and reversion unmated females. Additional log rank tests confirmed this conclusion (P=0.000001 for each comparison). Survivorships of the four reversion lines were homogenous (P=0.1962, Kruskal-Wallis test). After adjusting for multiple testing, survivorships of rev1 and rev3 lines were not different from survivorship of the control line, whereas survivorships of rev4 and rev5 lines were (P=0.1123, P=0.0491, P=0.0001, P=0.0001, respectively, Kruskal-Wallis test). Consequently, survival curves were not homogenous among control females and females of all reversion lines (P=0.0003, Kruskal-Wallis test), though mean and median lifespans of control females and females of all reversion lines were almost identical and varied between 53 and 55 days (Table 1). The average positive effect of mutation was approximately 10% of the control lifespan. Comparison of survival curves indicated that the mutation slowed aging slightly.

No difference was found between the lifespans of mutant and control males (Table 1, Figure 2E). The lifespan of males in two of four reversion lines, rev1 and rev3, was significantly different from the lifespan of mutant males (Table 1, Figure 2E) and control males according to the Kolmogorov-Smirnov test (P = 0.0063 for rev1; P = 0.0243 for rev3). These differences did not reflect an increase or a decrease in lifespan but fluctuations in the shape of survival curves (Figure 2E). Over the next two years, two other experiments confirmed the lack of difference in lifespan between mutant and control unmated males (Table 1, Figures 2F, 2G).

A significantly decreased lifespan was detected in mated mutant females compared to control females and females of the four reversion lines (Table 1, Figure 3A). To verify these results, the experiment was repeated and the lifespan of mutant females was again shown to be significantly lower than the lifespan of control females and females of all four reversion lines (Table 1, Figures 3B, 3C). Additional log rank tests for the combined results of the two experiments confirmed this conclusion (P=0.000001 for each comparison). Survivorships were homogenous among the four reversion lines (P=0.1198, Kruskal-Wallis test). Survivorships of all reversion lines were not different from survivorship of the control line (P=0.2542, P=0.3366, P=0.6245, P=0.4784, respectively, Kruskal-Wallis test), consequently, survivorships were homogenous among all five lines (P=0.2110, Kruskal-Wallis test). Experiments with mutant and control mated females were repeated four more times over three years. The last experiment was made simultaneously with reproduction assays. In all cases, the lifespan of mutant females was significantly lower than the lifespan of control females (Table 1, Figures 3D-G). In the last experiment, the difference between the lifespan of mutant and control females was greater than in the earlier experiments. Our results demonstrated a difference in lifespan between control and mutant mated females and between mutant and reversion unmated females. The average negative effect of mutation was approximately 15% of the control lifespan. Comparison of survival curves indicated that the mutation slightly accelerated aging.

After adjusting for multiple testing, the lifespan of mutant mated males was not different from the lifespan of control males and males with all four reversions (Table 1, Figure 3H). To verify these results, the experiment was repeated and identical results were obtained (Table 1, Figures 3I, 3J). Three other experiments confirmed the lack of difference between the lifespan of mutant and control mated males (Table 1, Figures 3K, 3L, 3M).

### Locomotion

**Figure 4:**
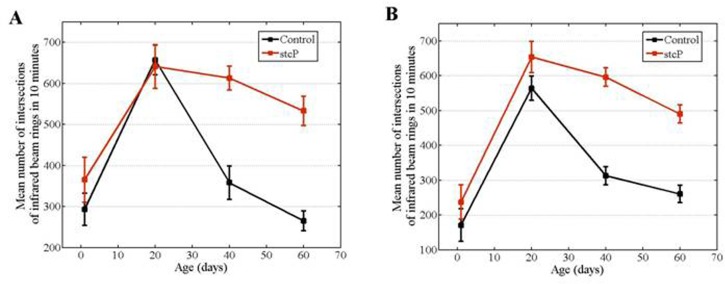
Age-dependent locomotion of mutant and control females. (**A**) Locomotion of unmated females in vials fixed in horizontal position. (**B**) Locomotion of unmated females in vials fixed in vertical position.

In both mutant and control unmated females, locomotor activity reached a maximum at 20 days of age and then decreased (Figure 4A). No difference in locomotion was detected in 1 day old or 20 day old unmated females, whereas locomotion of 40 day old and 60 day old mutant females was significantly higher than locomotion of 40 day old and 60 day old control females (Table 2, Figure 4A). Locomotion was also measured in 40-day-old unmated females from the rev3 line. No difference was detected compared to controls (Table 2).

**Table 2:** Parameters of female locomotion in the control, stcP, and rev3 lines

Direction of motion	Age of females, days	Line	No of vials ^1^	Mean No of intersections ^2^ (s. e.)	Median No of intersections	P values for comparisons with stcP line	
						t test	Kruskal-Wallis test
Horizontal	1	Control	16	171 (47)	116.5	0.3320	0.2662
		stcP	16	237 (49)	163.5		
	20	Control	20	564 (35)	536	0.1259	0.0909
		stcP	20	653 (45)	688.5		
	40	Control	17	313 (26)	315	**0.0001**	**0.0001**
		stcP	17	596 (27)	624		
		rev 3	14	366 (13)	355	**0.0024**	**0.0009**
	60	Control	14	260 (25)	240	**0.0001**	**0.0001**
		stcP	14	490 (26)	522		
Vertical	1	Control	16	293 (39)	313	0.2916	0.2912
		stcP	16	365 (55)	336		
	20	Control	20	657 (36)	664	0.8062	0.8181
		stcP	20	641 (53)	702		
	40	Control	17	358 (41)	330	**0.0001**	**0.0001**
		stcP	17	613 (29)	632		
	60	Control	14	265 (24)	254.5	**0.0001**	**0.0001**
		stcP	14	533 (36)	575.5		

1 Each vial contained 10 females.

2 Number of intersections of three infrared beam rings during 10 minutes.

Standard errors were calculated from variance among vials.

Significant P-values are in bold.

Flies naturally tend to move against gravity. We investigated if the *stc* mutation altered fly geotaxis independently of locomotion *per se*. Results were similar regardless of whether vials were fixed in horizontal and vertical positions, i.e. horizontal or vertical motion was primarily measured (Figures 4A, 4B). We did not use mechanical stimuli to induce movements, which might explain the lack of difference between the results. Thus, we considered the two variations of the experiment as replicates.

### Reproduction

**Figure 5:**
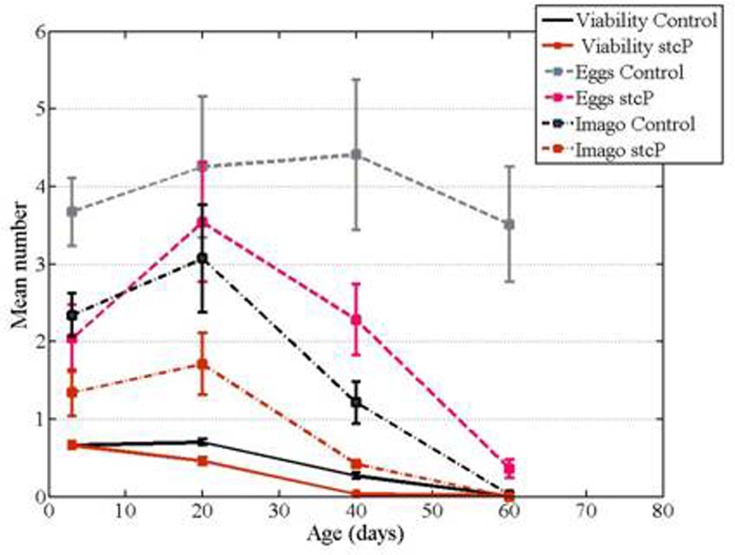
Age-dependent fecundity of mutant and control females and viability of their progeny. Eggs: the mean number of eggs per female. Imagoes: the mean number of imago offspring per female. Viability: egg to imago viability, the number of imago offspring related to the number of eggs laid.

The number of eggs laid by mutant females was significantly lower compared to controls at 3 and 40 days of age. The visual difference in the number of eggs laid by 20 day old females was substantial but not significant. The number of adult progeny was significantly lower from mutant females at 20 and 40 days of age compared to controls (Table 3, Figure 5). Almost no progeny were obtained from any 60 day old females, and the difference between mutant and control females was not detectable. Egg-to-adult viability of progeny from mutant females at 3, 40 and 60 days of age was also lower than controls (Table 3, Figure 5). Overall, reproductive ability appeared to be reduced in mutant females compared to controls.

**Table 3:** Parameters of female reproduction and viability of offspring in the control and stcP lines

Age of females, days	Line	No of vials	Mean (s. e.)	Median	P values for comparisons with stcP line	
					t test	Kruskal-Wallis test
**No of eggs per female**						
3	Control	20	3.67 (0.44)	3.20	**0.0122**	**0.0049**
	stcP	20	2.04 (0.43)	1.25		
20	Control	20	4.25 (0.91)	2.65	0.4711	0.7762
	stcP	20	3.54 (0.77)	2.85		
40	Control	20	4.41 (0.97)	3.70	**0.0069**	**0.0077**
	stcP	20	2.28 (0.46)	1.60		
60	Control	20	3.51 (0.74)	3.20	**0.0004**	**0.0001**
	stcP	20	0.36 (0.12)	0.11		
**No of imago offspring per female**						
3	Control	20	2.34 (0.28)	2.20	**0.0188**	**0.0097**
	stcP	20	1.34 (0.29)	0.75		
20	Control	20	3.07 (0.69)	1.8	**0.0190**	0.1711
	stcP	20	1.71 (0.39)	1		
40	Control	20	1.21 (0.27)	1	**0.0007**	**0.0001**
	stcP	20	0.42 (0.05)	0		
60	Control	20	0.02 (0.02)	0	NA	NA
	stcP	20	0	0		
**Egg to pupa viability**						
3	Control	20	0.674 (0.035)	0.688	0.6229	0.7571
	stcP	20	0.705 (0.051)	0.686		
20	Control	20	0.714 (0.037)	0.760	**0.0012**	**0.0015**
	stcP	20	0.485 (0.054)	0.516		
40	Control	20	0.276 (0.042)	0.250	**0.0001**	**0.0001**
	stcP	20	0.030 (0.014)	0		
60	Control	20	0	0	NA	NA
	stcP	20	0	0		
**Egg to imago viability**						
3	Control	20	0.655 (0.067)	0.685	0.9655	0.8286
	stcP	20	0.658 (0.047)	0.631		
20	Control	20	0.696 (0.036)	0.688	**0.0005**	**0.0005**
	stcP	20	0.460 (0.050)	0.523		
40	Control	20	0.267 (0.041)	0.250	**0.0001**	**0.0001**
	stcP	20	0.029 (0.014)	0		
60	Control	20	0	0	NA	NA
	stcP	20	0	0		

Standard errors were calculated from variance among vials.

Significant P-values are in bold.

### *stc* transcript amounts

**Figure 6:**
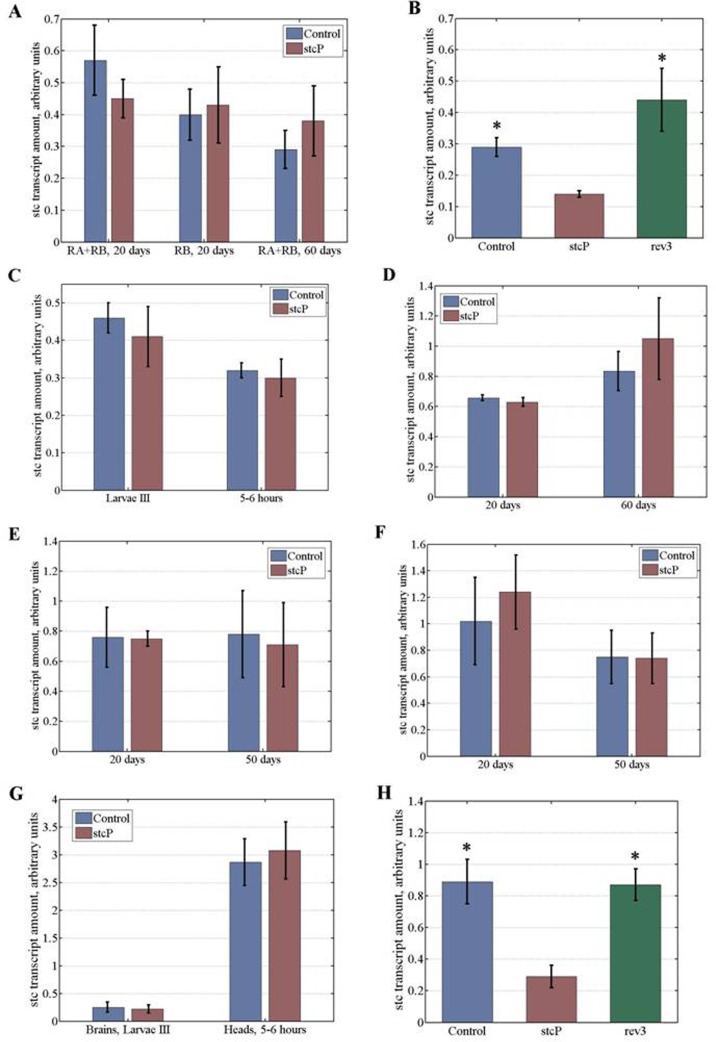
*Stc* transcript amounts at different stages of development and in flies of different age. Transcript amounts in (**A**) young and old unmated females. (**B**) embryos, experiment #1. (**C**) wandering stage III female larvae and 5 to 6 hour old virgin females. (**D**) young and old mated females. (**E**) ovaries of young and old unmated females. (**F**) ovaries of young and old mated females. (**G**) brains of wandering stage III female larvae and heads of 5 to 6 hour old virgin females. (**H**) embryos, experiment #2. Asterisks denote significant differences (P<0.05).

To understand the molecular basis of differences in lifespan, locomotion and reproduction caused by mutation, we assessed the effect of *stc^KG01230^* on *stc* transcript amounts (Table 4). Four annotated and two experimentally confirmed *stc* transcripts (RA and RB) that differ by 21 nucleotides due to alternative splicing are reported (http://flybase.org). We estimated total *stc* transcript amounts in 20 day old and 60 day old unmated females and males. No difference was found between control and mutant females (Table 4, Figure 6A) or control and mutant males (Table 4). The same results were obtained when comparing amounts of the sole RB transcript in 20 day old flies (Table 4, Figure 6A). Based on these results and the technical impossibility of measuring the sole RA transcript amount, we measured total *stc* transcript amounts in all further experiments, which were primarily restricted to females since *stc^KG01230^* did not affect male lifespan.

**Table 4:** *stc* transcript amounts in control, mutant and revertant lines

Source of RNA	Line	No of experiments	Mean (s. e.), arbitrary units	P values for comparisons with stcP line	
				t test	Kruskal-Wallis test
Embryos, 0-12 hours	Control	1	0.320 (0.02)*		
	stcP	1	0.224 (0.01)*		
Embryos, 12-18 hours	Control	1	0.333 (0.02)*		
	stcP	1	0.133 (0.03)*		
Embryos, 0-18 hours	Control	4	0.294 (0.003)	**0.0033**	**0.0209**
	stcP	4	0.140 (0.001)		
	rev3	4	0.444 (0.100)	**0.0273**	0.0833
Embryos, 0-18 hours	Control	4	0.894 (0.138)	**0.0073**	**0.0209**
	stcP	4	0.286 (0,066)		
	rev3	4	0.869 (0.097)	**0.0025**	**0.0209**
Female larvae, III stage	Control	5	0.459 (0.040)	0.5493	0.6242
	stcP	4	0.407 (0.077)		
Female larvae, III stage	Control	5	0.238 (0.029)	0.3305	0.2207
	stcP	4	0.191 (0.035)		
Females, 5-6 hours	Control	5	0.318 (0.021)	0.7415	0.8065
	stcP	5	0.300 (0.045)		
Unmated females, 20 days	Control	4	0.565 (0.110)	0.3977	0.5637
	stcP	4	0.449 (0.063)		
Unmated females, 20 days, RB	Control	2	0.401 (0.083)	0.8511	1
	stcP	2	0.433 (0.123)		
Unmated females, 60 days	Control	5	0.286 (0.064)	0.4960	0.6015
	stcP	5	0.376 (0.110)		
Mated females, 20 days	Control	4	0.658 (0.017)	0.5074	0.4624
	stcP	5	0.630 (0.034)		
Mated females, 60 days	Control	5	0.834 (0.126)	0.4645	0.6242
	stcP	4	1.048 (0.268)		
Ovaries, unmated females, 20 days	Control	2	0.757 (0.197)	0.9851	1
	stcP	2	0.747 (0.054)		
Ovaries, unmated females, 50 days	Control	2	0.782 (0.292)	0.9232	0.4386
	stcP	2	0.707 (0.275)		
Ovaries, mated females, 20 days	Control	2	1.019 (0.329)	0.8572	0.4386
	stcP	2	1.242 (0.284)		
Ovaries, mated females, 50 days	Control	2	0.749 (0.202)	0.9879	0.4386
	stcP	2	0.738 (0.194)		
Brain, female larvae, III stage	Control	5	0.251 (0.090)	0.7809	0.7540
	stcP	5	0.221 (0.069)		
Heads, females, 5-6 hours	Control	4	2.867 (0.420)	0.8848	0.3272
	stcP	5	3.074 (0.515)		
Unmated males, 20 days	Control	4	0.211 (0.036)	0.3113	0.2482
	stcP	4	0.162 (0.026)		
Unmated males, 20 days, RB	Control	2	0.207 (0.010)	0.1618	0.1213
	stcP	2	0.154 (0.019)		
Unmated males, 60 days	Control	2	0.513 (0.165)	0.7832	0.4386
	stcP	2	0.441 (0.162)		

Standard errors were calculated from the variance among biological replicates.

* Standard errors were calculated from the variance among technical replicates.

Significant P-values are in bold case.

The amount of RA and RB *stc* transcripts was measured in all cases except when sole RB transcript is specially indicated

*stc* is predominantly expressed in embryos. According to modENCODE Temporal Expression Data (http://flybase.org), the level of *stc* mRNA is rather steady throughout embryogenesis, with very high amounts observed at early stages, and high amounts observed up to 18 hours of development. Higher amounts of *stc* mRNA in early embryos may be explained by the presence of maternally derived transcripts [9]. In preliminary experiments, we evaluated *stc* transcript amounts in mutant and control 0 to 12 hour and 12 to 18 hour embryos. In both cases, amounts of *stc* transcripts appeared to be lower in stcP embryos, with the effect being less pronounced at early stages (Table 4). To confirm the difference between the lines, we measured *stc* transcript amounts in mutant and control 0 to 18 hour embryos. This large interval allowed us to obtain the overall characteristics of control and mutant embryos and to offset the possible uneven contribution of eggs of different stages. Amounts of *stc* transcripts were significantly lower in mutant embryos compared to controls (Table 4, Figure 6B).

We then measured *stc* transcript amounts in mutant and control wandering stage III female larvae, 5 to 6 hour old females, 20 day old and 60 day old unmated and mated females, ovaries of 20 day old and 50 to 60 day old unmated and mated females, brain of stage III female larvae, and heads of 5 to 6 hour old females. No differences were observed between control and mutant lines in larvae, 5 to 6 hour old virgin females, and in young or old adult females, both unmated and mated (Figures 6C, 6A, 6D). *stc* transcripts are abundant in ovaries, presumably because of high demand in embryos [9]. However, no differences were seen in *stc* transcript amounts between the ovaries of mutant and control females, regardless of age and mating status (6E, 6F). The presence of equal amounts of maternally derived transcripts in control and mutant embryos may mask a difference in embryonic transcription, which explains why *stc* transcript amounts are less different between control and mutant embryos at early embryonic stages. Considering that *stc* is required for development of the nervous system, we measured *stc* transcript amounts in brains of stage III larvae and heads of mutant and control females. No differences were found (Table 4, Figure 6G).

Differences in amounts of *stc* transcripts were found only in embryos and these differences disappeared as early as in female larvae. We repeated experiments with embryos and larvae and confirmed this result (Table 4, Figure 6H). Finally, we measured *stc* transcript amounts in embryos of a reversion line rev3and found they were significantly higher than in mutant embryos and did not differ from *stc* transcript amounts in control embryos (Table 4, Figures 6B, 6H).

### Synaptic activity

**Figure 7:**
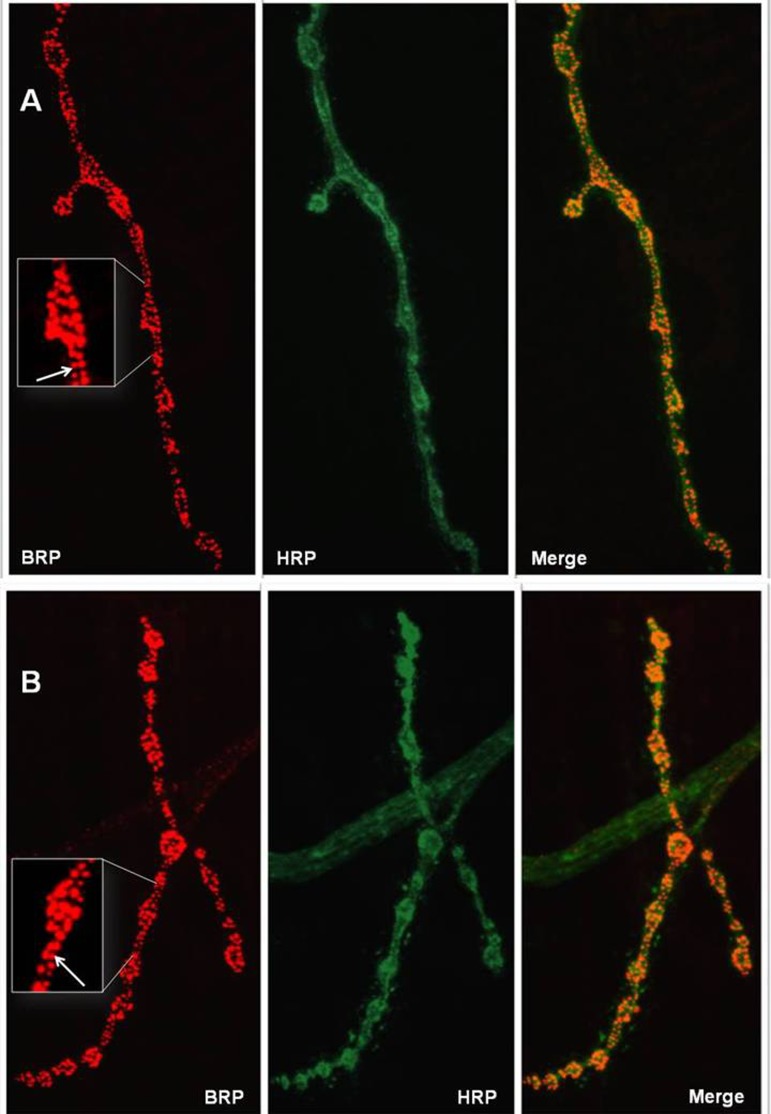
Active zones at representative neuromuscular junctions of wandering stage III female larvae. (**A**) control line. (**B**) stcP line. Left panels: NMJs are probed with anti-BRP (Bruchpilot), red color. Middle panels: NMJs are probed with anti-HRP (HorseRedish Peroxidase), green color. Right panels: merge. Enlarged parts of NMJs are shown in white frames. White arrows indicate red grains corresponding to active zones that were counted to characterize synaptic activity.

We investigated whether *stc^KG01230^* affected neuronal functions after the embryonic stage. No differences were observed in the number of synaptic active zones between female larvae that were mutants (124 ± 8) or controls (142 ± 12) (P = 0.2069 for Kruskal-Wallis test; Figure 7). Substantial differences in synaptic structure were also not detected.

## DISCUSSION

In this paper, we assessed the role of the neuronal gene *stc* in lifespan control using a control fruit fly line, a line with an insertional *stc^KG01230^* mutation, and four lines with precise reversions of this mutation, rev1, rev3, rev4, and rev5. Of note, precise reversions of mutations caused by insertions of P-element based vectors are uncommon [6]; we were fortunate to obtain four reversions, allowing us to make independent evaluations of their phenotypic effects. Lines rev3 and rev4 were obtained from different males that had delta 2-3 source of P element transposase in the genome; thus, they have resulted from two independent excisions of the *P{SUPor-P}* element. As expected, lifespans of lines rev1 and rev3 were statistically undistinguishable from lifespan of the control line. Minor differences could be explained by random uncontrolled fluctuations. Lines rev3 and rev4 were obtained from the same male that had delta 2-3 source of P element transposase in the genome; thus, they might have resulted from either two independent excisions of the *P{SUPor-P}* element or one premeiotic excision of *P{SUPor-P}*. Our results indicated that, most likely, premeiotic excision was the reversion mechanism. Indeed, survival curves for both unmated and mated rev4 and rev5 females were very similar and slightly but distinctly different from the survival curves of the control, rev1, and rev3 females (Figure 2D, 3C). Differences were specific and could not be explained by random uncontrolled fluctuations. In all lines with reversions, the normal *stc* gene structure was restored. In situ hybridization of a biotin labeled fragment of the P-element based vector comprising P-element inverted repeat and *white* sequences with salivary gland polythene chromosomes confirmed that in all reversion lines, no additional large insertions that could potentially affect lifespan appeared in the euchromatic part of the genome. We are not able to exclude a possibility that small fragments of the initial vector were inserted into new locations, though these events don't seen probable. It is virtually impossible to find these small insertions both by in situ hybridization with polythene chromosomes and by Southern blot analysis because a proper probe can't be selected. Overall, the nature of the differences among the reversion lines remains obscure. To add to the complexity, temporal fluctuations in lifespan of particular lines were observed. Changes in the mean lifespan of flies during long-term observations are not unusual and have been previously reported [12-14]. Hypothetically, they could be explained by some uncontrolled changes in the environment (magnetic field, sun activity, atmospheric pressure, etc). However, on the whole, in all lines with reversions, lifespan of the control line was also restored. This result confirmed the causal association between changes in lifespan and *stc^KG01230^* mutation.

It would be valuable to determine if other *stc* mutations have similar effects on fly longevity. However, only two insertion mutations affecting *stc* are publically available: *stc^KG01230^* used in this study and *stc^05441^* lethal mutation described in [8] and used in our previous work [5]. Even though the lethal phenotype of *stc^05441^* is typical for classic *stc* mutations, the insertion is actually located within an adjacent gene, *CG15269*. Besides, only heterozygotes can be used in lifespan assays. Finally, there is no proper control line for comparisons. Considering this, we decided that the study of this mutation will hardly add to our understanding of *stc* effects on longevity. The absence of proper control lines applies to all classic mutations as well. We are not aware of *stc* mutations available in private collections and do not expect that there are some, given that recently, there were no publications directly relating to *stc*. Lines for *stc*-specific RNAi knockdown are available, which can provide a decrease in *stc* transcript amounts similar to the effects observed in this study. However, none of the GAL4 driver lines available in stock collections governs expression only in embryos. Nevertheless, in future studies, we propose analyzing *stc*-specific RNAi knockdowns with several GAL4 drivers governing expression predominantly at the embryonic stage.

Only mutant females showed significant changes in lifespan compared to controls, with opposite effect seen in unmated and mated females. Sex specificity of the effect on lifespan confirmed our previous results [5], although in our earlier study, we used lines *y^1^ w^67c3^; P{SUPor-P}stc^KG01230^; ry* and *y^1^ w^67c23^*; *ry* initially obtained from the Bloomington Stock Center. In the initial genetic background, *stc^KG01230^* reduced lifespan both in unmated and mated females compared to controls. *w^1118^* has little or no effect on lifespan, whereas the *y^1^ w^67c23^* background appears to be long lived [15]. The difference in the lifespan effects of the *stc^KG01230^* mutation in unmated females compared to controls can be explained by differences in genetic background: in a long-lived background, the *stc* mutation decreased lifespan but had the opposite effect in a normal lifespan background. In mated females, the effect of the *stc* mutation on lifespan was similar. Overall, our results indicated unknown epistatic interactions. This conclusion agreed with our earlier data showing that the *stc* mutations had different effects on lifespan in different genetic backgrounds [5]. The genetic basis of these epistatic interactions remains to be elucidated, but our results support that *stc* interacts with other chromosomally unlinked genes that modify its effect on lifespan. Independent direct experiments indicate that naturally segregating genes interact epistatically with the “aging gene” *Sod* to modify its ability to extend longevity [16]. *stc* is expected to interact with other genes because it encodes a transcription factor and transcriptional cascades are recognized as a key regulatory mechanism [17]. Transcription factors such as FOXO, HSF-1, HIF-1 and others are crucial for the regulation of longevity and aging [for review, see 4].

Epistatic interactions might also be at least partially responsible for the sex-specific effects of *stc* on lifespan. Several groups of genes were identified that are regulated in a sex-biased manner under stress conditions. These range from expected differences in genes involved in reproductive physiology to those involved in amino acid utilization, sensory perception, immune response, and growth control. [18]. Given that many genes are involved in both longevity and stress resistance control, their sex-biased expression might account for the observed sex-specific patterns of longevity. In Drosophila, sex-specificity of lifespan control was reported earlier [19, 20], and was associated with insulin [21-23] and steroid signaling [24] and changes in protein homeostasis [25]. This sex-specificity may be based on the fundamental evolutionarily conserved systemic regulation of aging by the reproductive system [26].

In the experiments presented in this paper, unmated *stc^KG01230^* females lived longer than mated *stc^KG01230^* females (62±1 vs 50±2 days, averaged over all experiments). Reproduction is believed to shorten lifespan, and tradeoffs between lifespan and reproduction are widespread; however, increased lifespan and decreased reproduction can be uncoupled under certain conditions [for review, see 27]. Alteration of *stc* structure and function decreased both lifespan and fecundity of mated females, suggesting a direct correlation between survival and reproduction. *stc^KG01230^* flies had decreased viability compared to controls. This result suggested that the mutation was slightly deleterious overall even though it prolonged the lifespan of unmated females in certain backgrounds. It was shown that the female survival cost of mating is not associated with elevated feeding observed in females following mating [28]. Of all the traits, frequency of mating was significantly associated with the extent of the female survival cost of mating [28]. It remains to be assessed experimentally whether mating frequency is affected by *stc^KG01230^*. Another possibility is that *stc^KG01230^* effect on reproduction and survival is more specific and based on interactions with the metabolism of sex peptides. These male seminal fluid proteins can profoundly change female gene expression and physiology, egg production and frequency of mating [29]. Ii is also well known that mating increases the risk of infection. *Turandot M*, a member of a family of immune and stress response genes, provides survival benefits to females following sexually transmitted infections [30] and is another candidate for yet unknown interactions with *stc*.

Mutations in *stc* affect locomotion [31]. Our results confirmed this finding and showed a causal relationship between structural changes in *stc* and changes in locomotion. Locomotion was used as a marker of aging. General locomotor activity decreases with normal aging in animals and is often considered as a marker of aging [for review, see 32]. We assessed the effect of *stc^KG01230^* on locomotor activity in unmated females to determine if the mutation affects the rate of aging. A decline in locomotion was observed in both unmated mutant and unmated control females, however, a continued higher level of mobility in older mutant flies indicated slowed aging.. This confirmed our hypothesis that *stc^KG01230^* would slow aging in unmated females, based on initial comparison of survival curves.

The *P{SUPor-P}stc^KG01230^* construct was inserted into the untranslated region of *stc* and did not affect STC protein(s) (Figure 1A). However, this large insertion could affect transcription level and the molecular basis of *stc^KG01230^* effects on lifespan might have been associated with impaired gene expression. We hypothesized that age-dependent differences in the amount of *stc* transcripts would be observed between mutant and control female imagoes. However, we did not observe these hypothesized differences. Differences in particular organ(s) might have been masked when whole fly bodies were analyzed. *stc* expression is observed throughout developmental stages and in many organs, however, high *stc* transcription is limited to embryos and ovaries in females (http://flybase.org). We analyzed the amount of *stc* transcripts in ovaries of unmated and mated females of different ages and saw no differences between mutant and control flies. *stc* is involved in embryonic development of the nervous system [8] and is presumably necessary for its function later in life. However, no differences in *stc* transcript amounts were detected in the brain of mutant and control females. Also, according to our preliminary investigations, synaptic activity and structure were not changed in *stc^KG01230^* female larvae compared to controls. In future studies, we propose more deeply exploring the functional role of *stc* in the nervous system of larvae and imagoes.

*P{SUPor-P}stc^KG01230^* is a large insertion that might affect functions of genes located near *stc*. *CG15269*, a gene with unknown biological function, is located 388 bp upstream of the 5′ end of *stc*. *P{PZ}stc^05441^* insertion into *CG15269* was described in[8] as a *stc* mutation with typical phenotype. The nature of this phenomenon remains unknown and could be a subject of future studies that could also address the question whether the *P{SUPor-P}stc^KG01230^* insertion affects *CG15269* function. Several non-protein coding genes with unknown functions are located 1714 bp downstream of *stc* and further. At the moment, there is no data allowing imagine how the *P{SUPor-P}stc^KG01230^* insertion might affect their function. No other genes are found in the vicinity of *stc*, the nearest neighbors being *vasa* and *vasa intronic gene* located 39097 bp upstream of *stc* and *CG4168* located 43813 bp downstream of *stc*. Although we can't fully exclude remote effects of *P{SUPor-P}stc^KG01230^*, they don't seem probable.

We found that the *stc^KG01230^* mutation changed *stc* transcription only in embryos. This significant result was confirmed in two experiments, conducted over approximately 2 years. We were unable to separate female and male embryos to attribute our results to a particular gender. Our results in female larvae suggested that transcription might be still slightly lower in mutants (Figure 6C), whereas results in old females suggested that transcription might be slightly higher in mutants (Figures 6A, 6D). Although these differences were not significant, in the future we might study this question by increasing the resolution of our methods, to find causal association between age-dependent *stc* expression and lifespan. It is tempting, however, to suggest that lifespan might depend on gene function during early development. Accumulating data suggest that several key lifespan regulators such as mitochondrial electron transport chain enzymes, microRNAs, and the transcription factors HSF-1 and FOXO affect lifespan predominantly during early larval development and early adulthood [for review, see 4]. We hypothesize that the long-term, carry-over effects of the *stc* mutation might be epigenetically inherited in cell lineages. Alternatively, the STC transcription factor might participate in transcriptional cascades that predetermine structural and therefore functional properties of the adult nervous system. If embryonic transcription of *stc* and lifespan are causally associated, the exact mechanisms of this carry-over effect remains to be elucidated.

## MATERIALS AND METHODS

### Drosophila lines and crosses

The *y^1^ w^67c23^; P{SUPor-P}stc^KG01230^; ry* mutant line and the *y^1^ w^67c23^*; *ry* control line were from the Bloomington Stock Center and used in our previous work [5]. Standard substitution crosses with balancers and delta 2-3 source of P element transposase [10] were used to obtain lines with reversions of the *stc^KG01230^* mutation. Four lines with reversions of the marker *w^+^* phenotype were obtained from three males with the active transposase. In crosses, chromosomes 2 from control and mutant lines and chromosomes 2 with reversions were substituted into the X and chromosome 3 genetic background of *w^1118^* line to reduce the number of unnecessary mutations. In all experiments, flies were kept at 25°C on a standard medium of semolina, sugar, raisins, yeast and agar with nipagin, propionic acid and streptomycin.

### PCR and sequencing

DNA was extracted from 20 flies of each genotype using a standard phenol-chloroform method [33]. DNA was used in PCR reactions with primers pstc1 5′-GAACCGTTGCAGTACATTTAAC-3′ and pstc2 5′-GGAACAATCTCGAACTGCCC-3′ (expected product size 555 bp, Fig. 1A, B); pstc0 5′-CTAATTGGAAGGCGGAGCTC-3′ and pstc02 5′-CATTGAGAGTCCGGTGCTGT-3′ (2508 bp, Fig. 1A, C); pstc3 5′-ACACGTGTCTGGAGCTTTTCC-3′ and pstc4 5′-TCCGCTCTGTTACATAGCTGC-3′ (2562 bp, Fig. 1A, D). PCR products were sequenced with Big Dye Terminator V. 3.1. Kit (Applied Biosystems), according to the manufacturer's protocol on a ABI PRIZM 310 Genetic Analyser (Applied Biosystems). Six additional primers for sequencing were: 5′-TCCAACCAGACTGTCAAGTCAAATTAC-3′, 5′-TTCAATTAGCATGATCCAAGG-3′, 5′-AGACGTTGCTCTCGATCAGC-3′, 5′-AGACCACTCCCCGAAAACTG-3′, 5′-ATGTCAGCCCCTGTATGTGC-3′, 5′-AGAATCCAATCAGAGTGCGTC-3′.

### Tests for Wolbachia

Wolbachia was detected via real time quantitative PCR with primers to 16S rRNA gene, 5′-CATACCTATTCGAAGGGATAG-3′ and 5′-AGCTTCGAGTGAAACCAATTC-3′ [34].

### Lifespan assays

To assess the longevity of unmated flies, five virgin flies of the same genotype and sex, all collected on the same day from cultures with moderate density, were placed in replicate vials. To assess the longevity of mated flies, three virgin males and three virgin females of the same genotype were placed together in replicate vials. Flies were transferred to vials with fresh food containing approximately 5 mL of standard medium without live yeast on the surface weekly (virgin flies) or two times a week (mated flies). Dead flies were recorded daily. Experiments comparing fly lifespans were conducted simultaneously. Sample sizes were 60 to 100 flies/sex/genotype. All experiments were repeated three to six times. Lifespan was estimated for each fly as number of days alive from day of eclosion to day of death. Mean and median lifespan and survival curves were primarily used to characterize lifespan.

### Locomotion assays

Flies were collected and maintained as for lifespan assays but without recording deaths. Locomotion was measured in unmated females at age 1, 20, 40 or 60 days at the same time each day. Experiments comparing locomotion were conducted simultaneously. Sample sizes were 30 to 100 flies/genotype/age. One day before measurements, five flies of the same age and genotype were placed in replicate vials. To measure locomotor activity, vials were placed in a Drosophila Population Monitor (TriKinetics), either horizontally or vertically. Fly movement along the walls or in the middle of the vial interrupts infrared beam rings along the length of the vial. Beam interruptions are detected and counted electronically and totals were reported every five minutes to a host computer. Two measurements for five minutes were made for each vial. Locomotion was characterized as mean and median number of beam interruptions per vial.

### Fecundity and viability assays

Females aged 3, 20, 40 or 60 days were used. Sample sizes were 79 to 200 females/genotype/age. Fertilized females were placed in replicate vials, allowed to lay eggs for 12 hours and removed. Eggs were counted and transferred to fresh vials for development. Pupa and adult flies were counted in each replicate vial. Mean and median number of eggs and imagos per female were used to characterize fecundity. Mean and median egg-to-pupa and egg-to-imago viability per vial were used to characterize progeny viability.

### Larva dissection, immunostaining and microscopy

Third-stage larvae were dissected in phosphate buffered saline (PBS) and fixed in 4% formaldehyde at room temperature for 20 minutes. Preparations were incubated overnight at 4°C with primary antibodies NC82 (DSHB, USA) against Bruchpilot (BRP), a protein specific to active synaptic zones, and then incubated with secondary antibodies labeled by indocarbocyanine (Jackson Immunoresearch, USA) and with anti-HRP (HorseRadish Peroxidase) antibodies with fluorophore Alexa 647 (Jackson Immunoresearch, USA) at room temperature for two hours; placed in a medium for immunofluorescence VectaShield (Vector Labs, USA). Neuromuscular junctions were analyzed using the fourth muscle of the third and fourth abdominal segments of larvae using a confocal laser scanning microscope (LSM 510, Zeiss, Germany) at 63x magnification, 633 and 543 nm wavelength, and confocal slice thickness fixed at 1 micron. ImageJ and LSM Image Browser (Zeiss, Germany) were used to determine the number of synaptic active zones. Sample sizes were 13 to 15 larvae/genotype. Mean number of synaptic active zones was used to characterize synapse activity.

### Real-time RT-qPCR

Total RNA for real-time reverse transcription quantitative PCR (RT-qPCR) was extracted from 50 embryos aged 0–18 hours, from 20 female stage III female larvae, from 20 whole bodies of 5 to 6 hour old females, from 20 whole bodies of 20 day old and 60 day old virgin males and virgin and mated females, and from 50 pairs of ovaries of 20 day old and 50 day-old virgin and mated females, from 50 brains of stage III female larvae, from 30 heads of 5 to 6 hour old females using TRIzol reagent (Invitrogen) and DNase I Kit (TURBO DNA-free, Ambion) according to the manufacturers' instructions. Two to ten independent extractions/sex/mating status/genotype/age/tissue were made.

First-strand cDNA was synthesized using SuperScript II Reverse Transcriptase (Invitrogen) with oligo(dT) primers according to the manufacturer's instructions. Amounts of cDNA were determined by RT-qPCR using SYBR Green I/Rox in Chromo4 Real-Time PCR Detector (Bio-Rad).

*Gdh* and *Adh* housekeeping genes, characterized by relatively low expression comparable to *stc* expression were used as reference genes to normalize for differences in total cDNA between samples. Forward and reverse primer sequences were *stcRA+RB*: stc-rt1 5′-AACAGGCACAGCAACAACAA-3′ and stc-rt 2 5′-CCAGGGAGAAGTTAGTGTAGC-3′ (Figure 1A); *stcRB*: stc-RB1 5′-GGAGCCTTTGGACTGAACCC-3′ and stc-RB2 5′-ATTCGGAGATTGATGACTCAC-3′ (Figure 1A); *Gdh*: Gdh1 5′-TATGCCACCGAGCACCAGATTCC-3′ and Gdh2 5′-GGATGCCCTTCACCTTCTGCTTCTT-3′; *Adh*: Adhd3: 5′-CGGCATCTAAGAAGTGATACTCCCAAAA-3′ and Adhr3: 5′-TGAGTGTGCATCGAATCAGCCTTATT-3′.

MJ Opticon Monitor Analysis Software V. 3.1. 32 (Bio-Rad laboratories Inc., 2004-2005) was used to evaluate C(t) value. Intra-run calibrations were used for each panel of experiments, given the fact that the experiments were conducted for several years and two Bio-Rad PCR Detectors were used. Relative *stc* mRNA amount was considered as a measure of *stc* transcription level.

### Statistical analyses

To compare control, mutant and revertant lines, the Student's *t*-test was used for initial analysis of lifespan, locomotion, fecundity, viability, and transcript amount. Nonparametric, distribution-free Kruskal-Wallis test and Kolmogorov-Smirnov tests were used for further comparisons of all traits in lines of different genotypes. Bonferroni and false discovery rate [35] corrections for multiple analyses were used when appropriate.
